# A randomized trial of a mobile health intervention to augment cardiac rehabilitation

**DOI:** 10.1038/s41746-023-00921-9

**Published:** 2023-09-14

**Authors:** Jessica R. Golbus, Kashvi Gupta, Rachel Stevens, V.Swetha E. Jeganathan, Evan Luff, Jieru Shi, Walter Dempsey, Thomas Boyden, Bhramar Mukherjee, Sarah Kohnstamm, Vlad Taralunga, Vik Kheterpal, Susan Murphy, Predrag Klasnja, Sachin Kheterpal, Brahmajee K. Nallamothu

**Affiliations:** 1 Division of Cardiovascular Diseases, Department of Internal Medicine, University of Michigan, Ann Arbor, MI USA; 2 Michigan Integrated Center for Health Analytics and Medical Prediction (MiCHAMP), University of Michigan, Ann Arbor, MI USA; 3 Department of Internal Medicine, University of Missouri Kansas City, Kansas City, MO USA; 4 Department of Biostatistics, University of Michigan School of Public Health, Ann Arbor, MI USA; 5 Division of Cardiovascular Diseases, Department of Internal Medicine, Spectrum Health, Grand Rapids, MI USA; 6 School of Public Health, University of Michigan, Ann Arbor, MI USA; 7 CareEvolution, Ann Arbor, MI USA; 8 Departments of Statistics & Computer Science, Harvard University, Boston, MA USA; 9 School of Information, University of Michigan, Ann Arbor, MI USA; 10 Department of Anesthesiology, University of Michigan, Ann Arbor, MI USA; 11 The Center for Clinical Management and Research, Ann Arbor VA Medical Center, Ann Arbor, MI USA

**Keywords:** Cardiovascular diseases, Clinical trial design

## Abstract

Mobile health (mHealth) interventions may enhance positive health behaviors, but randomized trials evaluating their efficacy are uncommon. Our goal was to determine if a mHealth intervention augmented and extended benefits of center-based cardiac rehabilitation (CR) for physical activity levels at 6-months. We delivered a randomized clinical trial to low and moderate risk patients with a compatible smartphone enrolled in CR at two health systems. All participants received a compatible smartwatch and usual CR care. Intervention participants received a mHealth intervention that included a just-in-time-adaptive intervention (JITAI) as text messages. The primary outcome was change in remote 6-minute walk distance at 6-months stratified by device type. Here we report the results for 220 participants enrolled in the study (mean [SD]: age 59.6 [10.6] years; 67 [30.5%] women). For our primary outcome at 6 months, there is no significant difference in the change in 6 min walk distance across smartwatch types (Intervention versus control: +31.1 meters Apple Watch, −7.4 meters Fitbit; *p* = 0.28). Secondary outcomes show no difference in mean step counts between the first and final weeks of the study, but a change in 6 min walk distance at 3 months for Fitbit users. Amongst patients enrolled in center-based CR, a mHealth intervention did not improve 6-month outcomes but suggested differences at 3 months in some users.

## Introduction

Cardiac rehabilitation (CR) is an evidence-based, multidisciplinary secondary prevention program for patients with cardiovascular disease that includes physical activity and exercise training amongst its core components^1^. CR has been demonstrated to reduce mortality and hospital readmissions and improve both quality-of-life and functional capacity^2–6^. Despite these benefits, activity levels at CR completion may be suboptimal and patients have been shown to resume prior sedentary behaviors following program graduation, placing them at risk for recurrent events^7,8^.

In light of these gaps, there has been growing interest in leveraging mobile health (mHealth) technologies, such as smartphones and smartwatches, to support patients in achieving and then sustaining lifestyle behaviors related to exercise and physical activity first introduced during CR^9^. This can be done through use of text messaging and mobile application-based self-monitoring and engagement strategies. While promising, data are currently limited with most evidence derived from predominantly short-term, non-randomized studies that have focused on deploying mHealth technologies during center-based or virtual CR programs, and rarely included evaluations of intervention delivery after program graduation^10^. In addition, most interventions have been “static” in that they do not account for participants’ changing environment or circumstances. Just-in-time adaptive interventions (JITAIs) are a novel intervention design that leverage contextual information (e.g., weather) from wearable devices to deliver “in-the-moment,” tailored support to users in the form of push notifications to shape health behaviors^11^. Emerging data suggest adding such contextual information to motivational cues as part of an adaptive intervention may improve outcomes^12^.

Herein, we present the results of the Virtual AppLication-supported ENvironment To INcrease Exercise (VALENTINE) Study, a remotely administered, randomized clinical trial designed to test a mHealth intervention. The study tests the hypothesis that a mobile application and contextually tailored text messages delivered as a JITAI can augment physical activity levels for low and moderate risk patients enrolled in center-based CR and then support patients in maintaining increased physical activity levels over an extended follow-up of 6-months.

## Results

### Study population

**Fig. 1:**
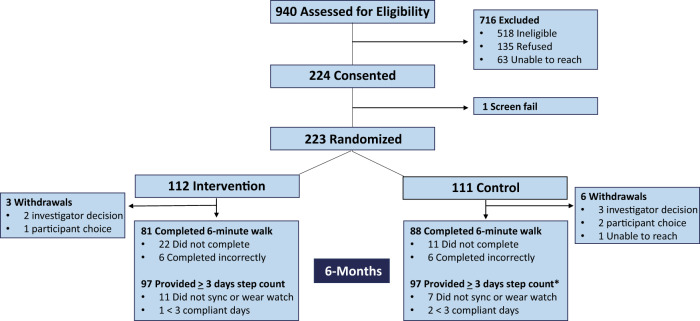
Consort diagram. *One participant withdrew from the study at 200 days due to hip fracture and was unable to complete his 6-month 6 min walk though did provide final step count data. Thus completed step count and withdrawals add to 112 control participants.

Between October 23, 2020 and March 25, 2022, 940 patients were screened for eligibility. Of these, 422 patients met study inclusion criteria with 223 (52.8%) randomized to the intervention (*n* = 112) or control (*n* = 111) groups (Fig. 1). Among randomized participants, 1 could not be reached after consent and was withdrawn while 2 were withdrawn after randomization due to failure to meet inclusion criteria (i.e., upcoming surgery, unsafe for home exercise per exercise physiology team). Thus, 220 participants were included in the primary analysis.

Baseline characteristics of participants showed no substantial differences between the two groups (Table 1). Participants had a mean age of 59.6 (10.6) years with 67 women (30.5%) and 184 White participants (83.6%). 175 participants (79.5%) enrolled in CR at University of Michigan Health and 45 (20.5%) at Spectrum Health. Most owned an iPhone and were provided with an Apple Watch [138 (62.7%)] for study participation. The most common indication for enrollment in CR was after coronary revascularization, primarily percutaneous coronary intervention [105 (47.7%)]. Participants enrolled 20.7 (SD 12.7) days after starting CR.

**Table 1:** Baseline characteristics of Study Population.

	Intervention	Control
Age, years	59.5 (10.7)	59.7 (10.5)
Sex, *N* (%)		
Male	75 (67.6%)	78 (71.6%)
Female	36 (32.4%)	31 (28.4%)
Race, *N* (%)		
Asian	6 (5.4%)	3 (2.8%)
Black	4 (3.6%)	8 (7.3%)
White	92 (82.9%)	92 (84.4%)
Other	9 (8.1%)	6 (5.5%)
Ethnicity, *N* (%)		
Hispanic Ethnicity	2 (1.8%)	1 (0.9%)
Non-Hispanic Ethnicity	104 (93.7%)	104 (95.4%)
Unknown	5 (4.5%)	4 (3.7%)
Study site, *N* (%)		
Michigan Medicine	90 (81.1%)	85 (78.0%)
Spectrum Health	21 (18.9%)	24 (22.0%)
Phone type, *N* (%)		
iPhone (Apple Watch Series 4)	70 (63.1%)	68 (62.4%)
Android phone (Fitbit Versa 2)	41 (36.9%)	41 (37.6%)
Indication for cardiac rehabilitation, *N* (%)		
Percutaneous coronary intervention	58 (2.3%)	47 (43.1%)
Coronary artery bypass grafting	21 (18.9%)	18 (16.5%)
Valve repair or replacement	22 (19.8%)	27 (24.8%)
Percutaneous coronary intervention or coronary artery bypass grafting & valve repair or replacement	3 (2.7%)	3 (2.8%)
Coronary artery disease or acute coronary syndrome, not revascularized	7 (6.3%)	14 (12.8%)
Body mass index, kg/m^2, a^	30.4 (6.5)	30.9 (7.3)
Comorbidities, *N* (%)		
Atrial fibrillation/flutter^a^	29 (26.1%)	22 (20.2%)
Coronary artery disease/Myocardial infarction	92 (82.9%)	81 (74.3%)
Diabetes mellitus^a^	31 (27.9%)	31 (28.4%)
Hypertension^a^	74 (66.7%)	68 (62.4%)
Heart failure	21 (18.9%)	17 (15.6%)
History of valve repair or replacement	29 (26.1%)	29 (26.6%)

^a^Data available for Michigan Medicine patients only.

### Study execution

Among 220 participants in the primary cohort, 6 participants withdrew from the study a median of 101 (range 41–200) days after enrollment: 2 in the intervention group and 4 in the control group (Fig. 1). Over the duration of the study, participants wore their watches for a median of 181 (IQR 174.8, 182.0) of a possible 182 days with 90% of participants wearing their watches for at least 75% of days. In a univariate regression analysis, wear time did not decline over the course of the study when measured as a continuous variable (*p* = 0.89). Participants in the intervention group of the study received a mean of 163.1 (SD 17.3) activity text messages and 82.9 (SD 9.4) exercise text messages over the duration of the study with a mean of 0.94 (SD 0.08) activity messages/day and 0.48 (SD 0.04) exercise messages/day. Ninety-three (93) participants in the intervention group completed the System Usability Scale (SUS) at 6-months with a mean score of 78.1 (SD 16.2).

### Primary end point

**Fig. 2:**
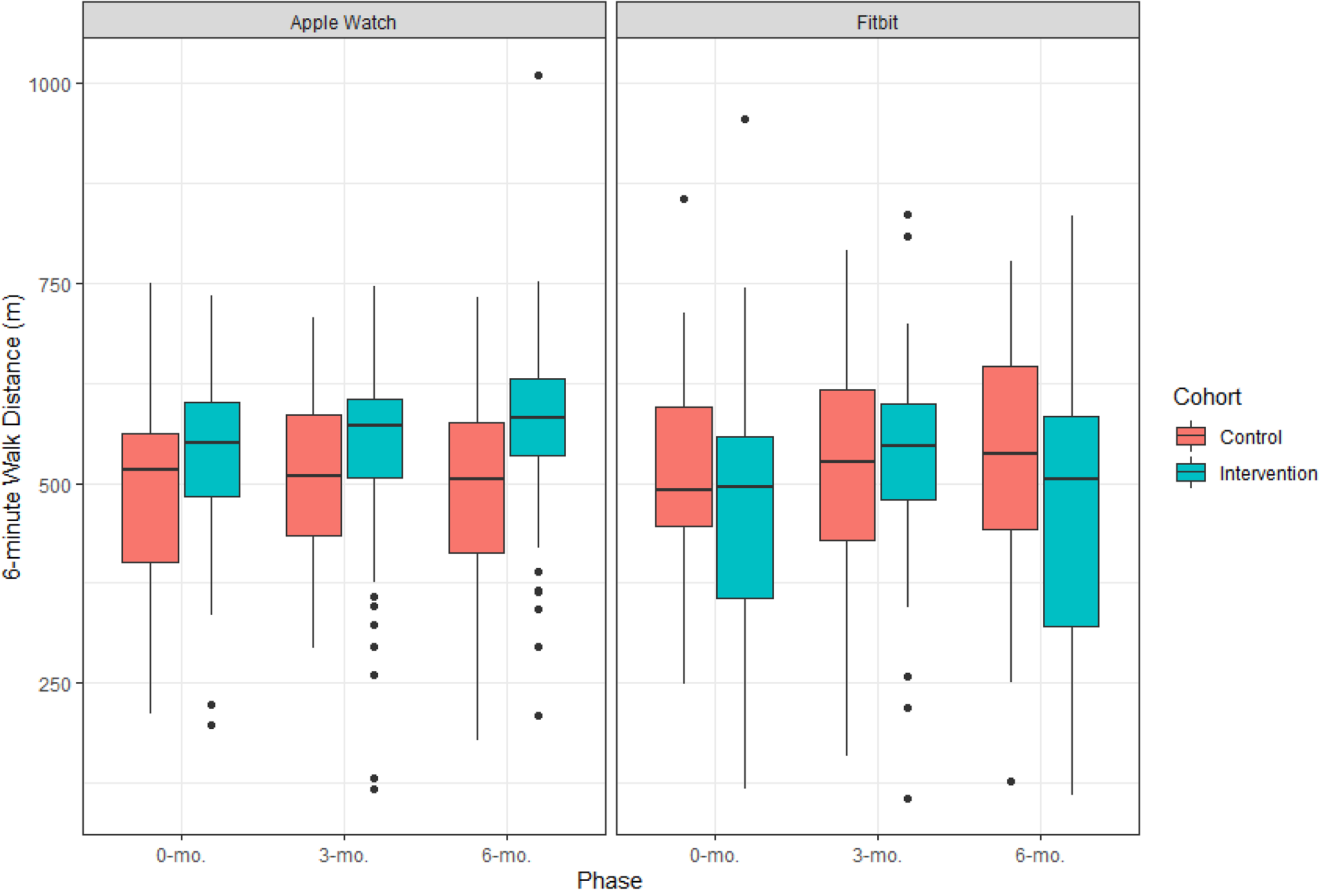
6-min walk distance at baseline, 3-months, and 6-months for intervention and control groups of the study by device type. The upper and lower bounds of the box refer to the 25th and 75th percentiles, and the line intersection in the box refers to the median. The dots outside of whiskers refer to outlying 6-min walk distances.

Overall, 6 min walk distance was available for 202 (91.8%) participants at baseline, 172 (78.2%) participants at 6-months, and 169 (76.8%) participants at both time points. Baseline 6-min walk distance was 492.1 (SD 122.5) meters for participants in the control group and 510.8 (SD 133.0) meters for participants in the intervention group (Supplementary Tables 1, 2). At 6-months, 6 min walk distance increased to 505.9 (SD 130.9) meters and 530.3 (SD 157.9) meters for the control and intervention groups, respectively (Supplementary Table 1). In a univariate regression analysis, mean change in 6 min walk distance for the intervention group as compared to the control group was + 31.1 meters for Apple Watch users and −7.4 meters for Fitbit users, which was not statistically significant (*p* = 0.28; Fig. 2, Table 2). Effects of the intervention on 6 min walk distance at 6-months were consistent across pre-specified subgroups of the population except for with respect to age in Fitbit users (Supplementary Fig. 1). In an exploratory multivariable regression analysis adjusting for baseline covariates, results were unchanged overall but suggested possible improvements in 6-minute walk distance in Apple Watch users ( + 41.0 meters Apple Watch, 95% CI 9.8–72.2; −11.4 meters Fitbit, 95% CI −77.6 to 54.9; *p* = 0.06 for joint t-test).

**Table 2:** Primary, Secondary, and Exploratory Outcomes.

	Outcome	Confidence Interval	*P*-Value
Primary Outcome			
Change in 6-min walk distance, 6-months, meters (Apple Watch)	+ 31.1	(− 8.0, 70.2)	0.28^a^
Change in 6-min walk distance, 6-months, meters (Fitbit)	− 7.4	(− 62.9, 48.2)	
Secondary Outcome			
Change in 6-min walk distance, 3-months, meters (Apple Watch)	− 8.5	(− 45.0, 28.0)	0.60
Change in 6-min walk distance, 3-months, meters (Fitbit)	+ 65.7	(16.2, 115.3)	0.03
Change in mean daily step count, 6-months, steps (Apple Watch)	+ 69.0	(− 879.4, 1017.2)	0.32^a^
Change in mean daily step count, 6-months, steps (Fitbit)	− 958.4	(− 2206.6, 289.8)	
Exploratory Outcomes			
Change in mean daily step count, 3-months, steps (Apple Watch)	359.4	(− 455.1, 1173.8)	0.59^a^
Change in mean daily step count, 3-months, steps (Fitbit)	298.1	(− 797.8, 1394.1)	
Change in EuroQol Visual Analogue Scale	1.8	(− 2.7, 6.3)	0.43

^a^*P*-values refer to the results of regression analyses to jointly test the null hypothesis of no change between baseline and 3-months or 6-months, respectively. If the null hypothesis of no effect was rejected than device-specific estimates of statistical significance were determined, and these *p*-values displayed.

Changes in activity and quality-of-life measures refer to intervention group compared to the control group.

### Secondary outcomes and other prespecified analyses

**Fig. 3:**
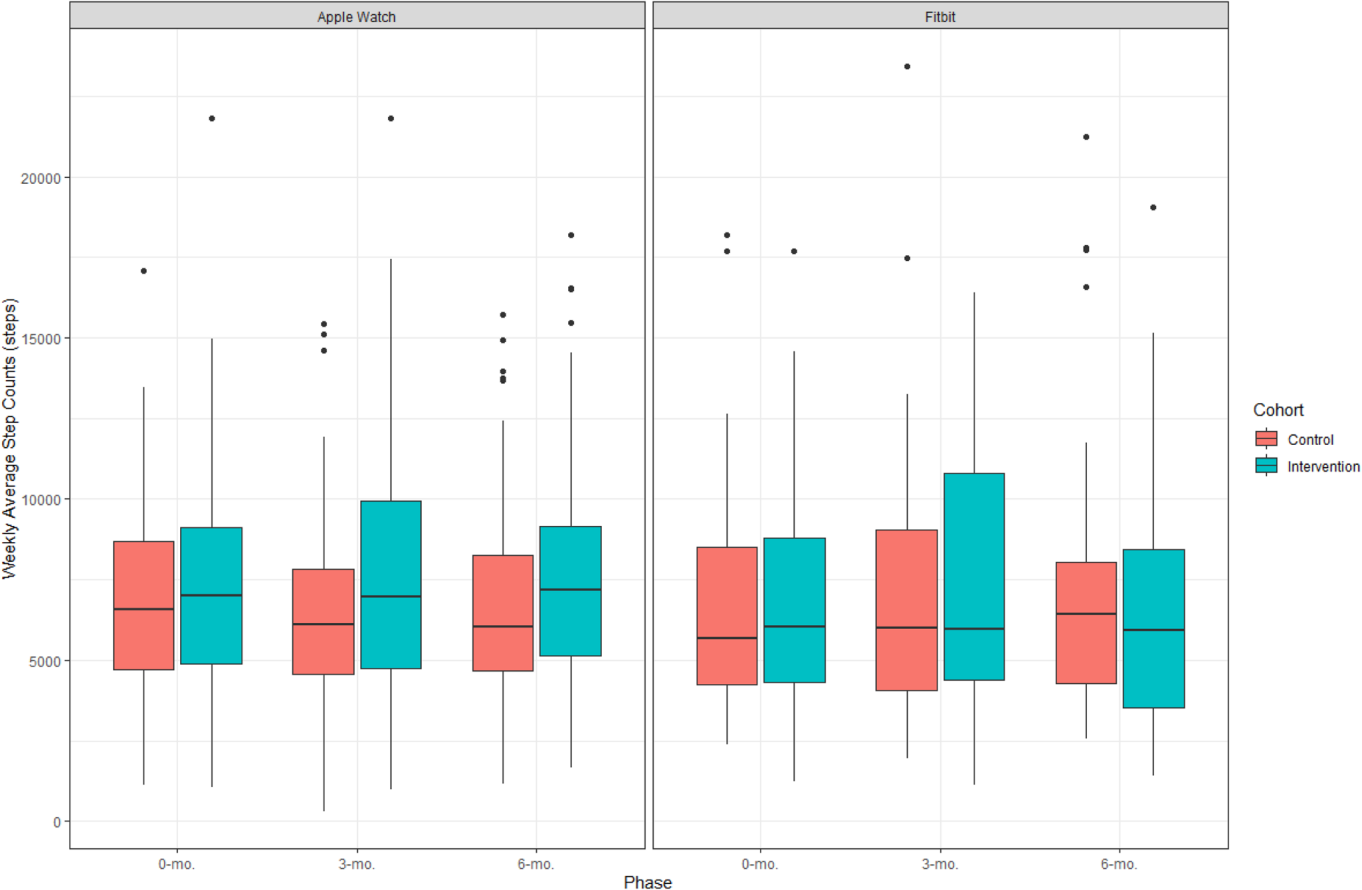
Step Count at baseline, 3-months, and 6-months for intervention and control groups of the study by device type. The upper and lower bounds of the box refer to the 25th and 75th percentiles, and the line intersection in the box refers to the median. The dots outside of whiskers refer to outlying step counts.

At 3-months, 6-min walk distance increased for both the control and intervention groups of the study with mean distances of 505.4 (SD 135.3) meters and 531.2 (SD 137.6) meters, respectively (Supplementary Table 1). In a univariate analysis, mean change in 6-min walk distance for the intervention group as compared to the control group was statistically significant for Fitbit users at + 65.7 meters (95% CI 16.2–115.3; *p* = 0.03) but not for Apple Watch users at −8.5 meters (95% CI −45.0 to 28.0; *p* = 0.60) (Fig. 3, Table 2).

Step count was available for 218 (99.1%) participants at baseline, 195 (88.6%) participants at 6-months, and 194 (88.2%) participants at both time points (Supplementary Table 1). Baseline step count was 6911.4 (SD 3190.1) steps for participants in the control group and 7294.9 (SD 3665.0) steps for participants in the intervention group. At 3-months, in a univariate analysis, step count increased for participants in the intervention group [7652.9 (SD 3918.2) steps] but decreased for the control group [6636.4 (SD 3671.1)] (*p* = 0.05 for comparison of 3-month step count) though the change in step count from baseline to 3-months was not significantly different between the two groups (intervention versus control group: + 359.4 steps Apple Watch, 95% CI −455.1 to 1173.8; + 298.1 steps Fitbit, 95% CI −797.8 to 1394.1; *p* = 0.59). In univariate analyses, there was no significant difference in change in step count (intervention versus control group: + 69.0 steps Apple Watch, 95% CI −879.4 to 1017.2; −958.4 steps Fitbit, 95% CI −2296.6 to 289.8; *p* = 0.32) or change in quality-of-life (intervention versus control group: + 1.8, 95% CI −2.7 to 6.3; *p* = 0.43) at 6-months (Table 2).

## Discussion

In this randomized clinical trial of low and moderate risk patients enrolled in center-based CR who were provided a smartwatch, we found an mHealth intervention consisting of a mobile application and contextually-tailored text messages did not improve 6-month outcomes as assessed by remote 6-min walk distance. However, we noted preliminary findings in secondary and exploratory analyses that could indicate lessons for future work. First, we found intervention group participants provided with a Fitbit experienced modest improvement in 6-min walk distance which exceeds established distances for a clinically meaningful change^13,14^. We also noted possible improvements in step counts at 3-months or when adjusting for differences in baseline covariates at 6-months. Yet these additional analyses should be interpreted cautiously as they were performed *post hoc*. Overall, our findings suggest that the intervention did not have a long-term impact on physical activity that was sustained over time but may have intermediate or potentially device-specific effects.

While CR reduces cardiovascular morbidity and mortality^3–5,15^, physical activity levels achieved at the end of the program may be suboptimal for some patients and may decline after program graduation for others^7,8^. One promising approach for improving physical activity has been through the use of mHealth technologies, and their application to CR has shown promising results, typically on short-term outcomes at 3-months^10^. Limitations, however, include their short duration of follow-up and use of static rather than dynamic intervention designs, failing to account for users changing circumstances and environment. Additionally, fewer studies have focused on using mHealth technology to extend the benefits of CR and, for those studies that have, results have been mixed, in part driven by varying intervention designs and comparison groups and small sample sizes^16–18^.

Our study aimed to address that gap by delivering a multifaceted mHealth intervention within the rigorous study design of a randomized clinical trial. As such, it adds unique data that should inform the debate around the use of mHealth technologies and complex interventions delivered through use of text messages and mobile applications. Several of its strengths should be highlighted. First, we utilized an inclusive study design that broadened the types of participants we included as compared with other mHealth studies. We enrolled patients owning both Apple and Android smartphones and provided them with a compatible smartwatch, increasing the relevance of our study findings to broader populations. In general, mHealth studies have focused on evaluating these devices in isolation, but the growing community of both users requires real-world studies like the VALENTINE Study to examine broader populations of users. Second, we delivered the trial remotely, allowing us to enroll patients from CR centers that included both an academic medical center and large community-based health system. This allowed us to include a broader set of participants with over 20% recruited from the community medical center that serves patients living in more rural areas.

However, this study should be interpreted in the context of the following potential limitations. First, it is important to note that we provided all participants with a smartwatch, including those enrolled in the control group of the study. Wearable activity trackers have been previously shown to increase physical activity levels in the general population^19^. In CR specifically, mHealth technology has been associated with adherence to center-based CR^20^ and its benefits^16,21^. Our findings indicate that the mHealth intervention was unsuccessful, but does not mean that smartwatches like the Fitbit or Apple Watch may not be useful when compared with no device. An indication of this possibility was that physical activity levels were higher in our control group than predicted, perhaps reflecting the impact of the smartwatch on physical activity levels given we lacked a control group without a smartwatch. Second, there are limitations of a remote 6-min walk distance as a measure of functional capacity. While a remote 6-minute walk test has been previously shown to be accurate^22^, other studies have drawn into question its value^23^. We thus additionally evaluated other related measures of physical activity such as step count. This will need to be further explored in future work. Third, loss to follow-up was higher than anticipated leaving us underpowered for our primary outcome. Fourth, text messages were contextualized though not necessarily personalized, aside from a subset of notifications including participants’ names. It is unknown if the intervention would have been more effective if text messages were further personalized based on additional factors (e.g., baseline physical activity). Finally, there were limitations related to the population enrolled. All participants were required to own a compatible smartphone, complete at least 2 center-based CR sessions, and be deemed low or moderate risk. Enrolled participants were also predominantly White and younger than the larger population of CR participants. Thus, whether the VALENTINE Study results can be generalized to patients not enrolled in center-based CR, those without a smartphone, or to other clinical and demographic groups remains unknown.

In conclusion, amongst patients enrolled in CR who were routinely provided a smartwatch, a mHealth intervention did not improve 6-month outcomes. Whether individual intervention components were effective in increasing physical activity requires further analysis. Future studies are warranted to determine how to use mHealth technology to optimally support users long-term in achieving and maintaining optimal cardiovascular health.

## Methods

### Overview

The VALENTINE Study is a remotely administered, randomized trial (ClinicalTrials.gov NCT04587882, registration date 10/14/2020)^24^. Participants in both groups received a smartwatch and usual care in the form of CR. Participants in the intervention group received a comprehensive mHealth intervention designed to augment and extend the benefits of CR. The authors are solely responsible for the design and conduct of this study, all analyses, drafting and editing of the paper, and its final contents. The study was approved by the University of Michigan Health IRB (HUM00162365).

### Participants, consent, and enrollment

Enrollment occurred from October 23, 2020 to March 25, 2022, recruiting low and moderate risk patients enrolled in center-based CR as guided by the American Association of Cardiovascular and Pulmonary Rehabilitation criteria^25^. Eligible patients were 18–74 years old who completed at least two CR sessions at two healthcare systems (University of Michigan Health and Spectrum Health) and spoke English. Patients were required to own either an Android-based phone or Apple iPhone with a study supported operating system. Full inclusion and exclusion criteria are available in Supplementary Table 3. Patients were excluded if they were unable to safely exercise without supervision or had at least one high risk condition (Supplementary Table 4).

All study activities occurred remotely. Participants were guided by study staff through a remote consent process and signed consent forms within the mobile study application MyDataHelps (CareEvolution, LLC). Consented participants were mailed an Apple Watch Series 4 or Fitbit Versa 2 in accordance with smartphone ownership, although they were given the option of using their own smartwatch if compatible. Randomization was performed by study staff using a permuted block design with variable block sizes of 2 to 6 and stratified by smartwatch (Apple Watch or Fitbit) using a random sequence computer-generated program. Neither participants nor study staff were masked to randomization assignment. Following consent, participants underwent a remote enrollment process at which time they were oriented to the mobile study application and assisted by study staff with pairing their devices and configuring their smartwatches to suppress notifications from other health and wellness applications.

### Study Ggroups

All participants received a smartwatch for outcome assessments and usual care in the form of CR. Participants were followed for 6-months. The study intervention has previously been described in full^24^. In brief, the intervention had four components. First, participants received two types of text messages (both one-way text messages): activity messages and exercise planning messages (Supplementary Table 5). Text messages were designed using conceptual behavioral health theories, including goal setting and implementation intentions^26–28^. Activity messages encouraged low level physical activity and incorporated the following contextual information: weather (i.e., temperature, precipitation), time of day (i.e., morning, lunch, afternoon, evening), day of week (i.e., weekend versus weekday), and duration within the study, correlating with phase in CR (ie, initiation [0–30 days], maintenance [31–120 days], completion [121–182 days] phases). Participants had a 25% probability of receiving an activity message at one of four time points each day. Exercise planning messages encouraged participants to exercise the next day within their target heart rate zones and were tailored to the season and duration within the study. Participants had a 50% probability of receiving an exercise message each evening. Exercise planning messages were initially written by CR exercise physiologists with the goal of capturing their voice and extending the human connections developed in center-based CR. Both exercise planning and activity messages could include additional features such as personalization with a participant’s preferred name, loss- or gain-framing, and inclusion of an emoji or hyperlink to the study dashboard. Participants selected the times at which to receive text message times during enrollment visits, with delivery delayed until day 8 of the study, though were allowed to modify selected times during the study if requested.

Participants in the intervention group received three additional components: 1) access to mobile application features that allowed them to engage in activity self-monitoring and goal-setting (Supplementary Fig. 2); 2) a weekly email summary of their smartwatch physical activity data; 3) smartwatch data provided to their exercise physiologists by email and through a web-based dashboard. Participants in the control group had access to either the Apple Health application or the Fitbit application, respectively, and downloaded the study mobile application to enable informed consent, data collection for outcomes assessments, and survey completion; however, it provided no additional functionality. Thus, participants in the control group did not have access to self-monitoring through the mobile application, tailored text messages, or weekly email summaries of their physical activity.

Participants in both groups were followed until study completion or withdrawal or termination by the study team. An independent medical monitor adjudicated all serious adverse events in a blinded manner according to prespecified definitions.

### Study outcomes

The primary outcome was change in 6-min walk distance from baseline to 6-months as measured remotely using the mobile study application and smartwatch. 6-minute walk distance was selected as it has prognostic significance in diverse cardiovascular disease populations, improves in response to exercise-based interventions, and can be accurately measured in a remote manner^22,29–32^. Participants were given verbal instructions during their enrollment appointments and written instructions in the mobile application on proper completion of the 6-minute walk test, which they then performed independently. For iPhone/Apple Watch and Android/Fitbit users, 6-min walk distance was calculated differently. For iPhone users, during their 6-min walk test, the mobile application triggered their Watch to enter workout mode and, as a result, the Watch recorded the distance walked at more frequent intervals. The mobile application then recorded the start and end timestamps corresponding to 6-minutes and added the distances during the 6-minute interval to determine distance walked. In contrast, for Android users, the Android phone recorded distance walked by using the phone’s Global Positioning System (GPS). Participants were given up to 30-days to complete 6-minute walk tests, but most completed them within 7 days. Distances less than 100 meters were excluded a priori from analyses given invalid data concerns, although the task was reassigned if it was determined to be invalid and within 30-days of its originally assigned date (Supplementary Table 5).

We had two additional secondary outcomes. First, we analyzed the change in mean daily step count between the first 7-days of the study prior to intervention receipt and the final 7-days of the study. For this analysis, participants were required to wear their watches for ≥ 3 days each week to be included in the analysis as at least 3 days of watch wear was felt to be necessary to capture a representative sample of daily activity. We excluded a priori from the analysis days that participants took fewer than 100 steps as they were presumed to have not worn their watches on those days, consistent with recent mHealth studies^33^. Participants in the intervention group of the study did not receive the intervention until week 2, allowing both groups to have a 1-week baseline period following enrollment (i.e., 175 day intervention period). Second, we evaluated change in 6-min walk distance from baseline to 3-months. Exploratory outcomes included: 1) Change in 6-min walk distance at 6-months adjusted for differences in baseline covariates based on recent guidance from the FDA and others in analyses of randomized controlled trials;^34–36^ 2) change in quality-of-life scores over 6-months as measured by the EuroQol visual analog scale (EQ-VAS); and 3) change in mean daily step count between the first 7-days of the study and 3-months (i.e., days 91–97). At the end of the study, participants in the intervention group completed a modified version of the SUS using a 6-point Likert scale to provide quantitative feedback on their experiences interacting with the mobile intervention^37^.

### Statistical analysis

Sample size calculations were based on change in 6-min walk distance at 6-months. The trial was designed to have approximately 80% power to detect a difference between the intervention and control groups irrespective of device type using a 2-sided significance level of 0.05 and change in 6-min walk distance of 50 meters as a change in 6-minute walk distance of 50 meters or less is clinically significant in most disease states^13,14^. We assumed a baseline 6-min walk distance of 400 meters and a standard deviation of 125 meters. Given a 10% drop-out rate, we planned to enroll 220 participants in total across the two arms.

All analyses were performed as a modified intention-to-treat analysis using data from participants with complete data for either the primary or secondary outcomes, respectively. Baseline clinical characteristics are described as means and standard deviations (SD) for continuous symmetric variables and median with interquartile range for skewed continuous variables. Categorical variables are presented as counts and percentages. We performed student t-tests for bivariate comparisons between continuous variables and Chi-square tests for comparisons across categorical variables. To account for known measurement differences between Fitbit and Apple devices, we performed a regression analysis to jointly test the null hypothesis of no effect between baseline and 6-months for 6-min walk distance (i.e. H_0_: β_0_(Fitbit) = β _0_ (Apple) = 0 where β_0_ refers to the estimated coefficients in the model for each device type, respectively). Such a test produces a single *p*-value inclusive of both device types, although it allows for separate device-specific effect sizes. The decision to do this was to conservatively estimate overall effects of the intervention, and this decision was determined a priori before any statistical analyses were conducted. A subsequent analysis was then performed to determine whether to reject the individual null hypotheses for the devices separately and, if the null hypothesis was rejected, device-specific estimates of statistical significance were determined. Subgroup analyses were performed for the primary outcome based on sex, age < 65 or ≥ 65, heart failure diagnosis, and study site.

Subsequently we performed an exploratory analysis for our primary outcome of change in 6-min walk distance at 6-months that performed an adjustment for differences in baseline covariates. In this analysis, we accounted for baseline covariates of age category ( < 65 or ≥ 65), sex, the presence of heart failure, study site, and baseline 6-min walk distance. As above, we tested the null hypothesis of no effect between baseline and 6-months for 6-minute walk distance. Finally, the SUS was scored using a modified scoring system with each item’s score contribution ranging from 0 to 5^37^. For odd questions, the score contribution was the scale position and for even questions the contribution was 5 minus the scale position. Scores were multiplied by 2 to obtain a measure of overall system usability with scores ranging from 0 to 100. For all analyses, the level of significance was set at *p* < 0.05.

### Supplementary information

Supplemental material

## Data Availability

The datasets used and/or analyzed during the current study are available from the corresponding author on reasonable request.

## Code availability

The underlying code for this study is available on Github (https://github.com/WIRED-L-um/VALENTINE-RCT) and shared with qualified researchers on reasonable request from the corresponding author.
