# Supplementary material for: A randomized trial of a mobile health intervention to augment cardiac rehabilitation
Source: NPJ Digit Med. 2023 Sep 14;6:173. doi: 10.1038/s41746-023-00921-9 (PMC10502072; doi:10.1038/s41746-023-00921-9)

A Randomized Trial of a Mobile Health Intervention to Augment Cardiac Rehabilitation

Jessica R. Golbus MD, MS,[1,2] Kashvi Gupta MBBS, MPH,[3] Rachel Stevens BS,[1] V.Swetha E. Jeganathan, MBBS, MRCS (Ophthal),[1] Evan Luff MS,[1] Jieru Shi MS,[4] Walter Dempsey PhD,[4] Thomas Boyden MD, MS,[5] Bhramar Mukherjee PhD,[6] Sarah Kohnstamm MD,[1] Vlad Taralunga BSE,[7] Vik Kheterpal MD,[7] Susan Murphy PhD,[8] Predrag Klasnja PhD,[9] Sachin Kheterpal MD, MBA,[10] Brahmajee K. Nallamothu MD, MPH[1,2,11]

1. Division of Cardiovascular Diseases, Department of Internal Medicine, University of Michigan, MI
2. Michigan Integrated Center for Health Analytics and Medical Prediction (MiCHAMP), University of Michigan, MI
3. Department of Internal Medicine, University of Missouri Kansas City, Kansas City, MO
4. Department of Biostatistics, University of Michigan School of Public Health, Ann Arbor, MI
5. Division of Cardiovascular Diseases, Department of Internal Medicine, Spectrum Health, MI.
6. School of Public Health, University of Michigan, Ann Arbor, MI, USA
7. CareEvolution, Ann Arbor, MI, USA
8. Departments of Statistics & Computer Science, Harvard University, Boston, MA, USA
9. School of Information, University of Michigan, Ann Arbor, MI, USA
10. Department of Anesthesiology, University of Michigan, MI.
11. The Center for Clinical Management and Research, Ann Arbor VA Medical Center, MI

Table of Contents:

- Page 3: Supplementary Tables 1-6
- Page 9: Supplementary Figures 1-2

Supplementary Table 1: Remotely Assessed 6-minute Walk Distance and Step Count at Baseline, 3-Months, and 6-Months from Study Enrollment

	Control		Intervention		P-value
	N	Mean (SD)	N	Mean (SD)	
**6-minute walk distance, meters**					
Baseline	101	492.1 (122.5)	101	510.8 (133.9)	0.30
3-months	93	505.4 (135.3)	89	531.2 (137.6)	0.20
6-months	89	505.9 (130.9)	83	530.3 (157.9)	0.27
**Mean daily step count, steps**					
Baseline	108	6911.4 (3190.1)	110	7294.9 (3665.0)	0.41
3-months	104	6636.4 (3671.1)	105	7652.9 (3918.2)	0.05
6-months	97	6976.6 (3780.9)	98	7395.9 (3859.6)	0.44

Student's t-test was performed to test differences in 6-minute walk distance and step count between intervention and control groups at each of the three times points.

Supplementary Table 2: Remotely Assessed 6-Minute Walk Distance and Step Count by Smartwatch Type at Baseline, 3-Months, and 6-Months from Study Enrollment

	Control				Intervention			
	Apple Watch		Fitbit		Apple Watch		Fitbit	
	N	Mean (SD)	N	Mean (SD)	N	Mean (SD)	N	Mean (SD)
**6-minute walk distance, meters**								
Baseline	64	485.6 (110.8)	37	503.4 (141.5)	64	530.7 (109.6)	37	476.5 (164.1)
3-months	60	507.9 (107.1)	33	500.7 (177.4)	59	534.5 (130.0)	30	524.9 (153.6)
6-months	59	496.6 (119.1)	30	524.0(152.2)	55	566.9 (121.7)	28	458.3 (194.8)
**Mean daily step count, steps**								
Baseline	68	6971.0 (2998.7)	40	6809.9 (3529.3)	69	7521.8 (3641.2)	41	6913.1 (3718.2)
3-months	67	6430.5 (3223.2)	37	7009.3 (4392.2)	68	7625.3 (3841.2)	37	7703.6 (4109.1)
6-months	62	6668.2 (3265.5)	35	7522.9 (4555.4)	62	7590.7 (3592.3)	36	7060.3 (4314.1)

Supplementary Table 3: Study Inclusion and Exclusion Criteria

Inclusion criteria.

1. Enrolled in cardiac rehabilitation at a study site
2. Age ≥ 18 and < 75 years
3. Owns an Android or iPhone with study supported operating software.
4. Understands English to enable informed consent, completion of study-related surveys, and compliance with study messages.
5. Enrolled in cardiac rehabilitation within the past 8-weeks based on one of the following indications and has completed at least 2 cardiac rehabilitation sessions:
    - Following percutaneous coronary intervention or coronary artery bypass grafting
    - Valve repair or replacement (either surgical or percutaneous)
    - Coronary artery disease/acute coronary syndrome not requiring revascularization

Exclusion Criteria:

1. Orthopedic or neurological condition limiting ability to exercise
2. Greater than mild cognitive impairment
3. Wrist too large to wear a smartwatch comfortably.
4. Enrolled in hospice care
5. Severe valvular stenosis or regurgitation
6. Unrevascularized, obstructive left main or proximal left anterior descending coronary artery disease
7. Exercise-induced ventricular tachycardia
8. Cardiac arrest within the prior 6 months
9. New York Heart Association class III or IV heart failure
10. Pulmonary arterial hypertension treated with inhaled or intravenous pulmonary hypertension-specific therapy
11. Ejection fraction <40%
12. Unsafe for participation as determined by a clinical nurse or investigative team

Supplementary Table 4: Reasons for Study Exclusion

Reasons for Study Exclusion	N[a]
1. Age <18 or $\geq$ 75	234
2. Not a Michigan Medicine or Spectrum Health patient with data available in the electronic medical record after January 1, 2013	5
3. Did not enroll in cardiac rehabilitation within past 8 weeks or did not complete at least 2 cardiac rehabilitation sessions based on a study-qualifying diagnosis	198
4. Orthopedic or neurological condition limiting ability to exercise	71
5. Greater than mild cognitive impairment	3
6. Enrolled in hospice care	0
7. Severe valvular stenosis or regurgitation	8
8. Unrevascularized, obstructive left main or proximal left anterior descending coronary artery disease	7
9. Exercise-induced ventricular tachycardia	4
10. Cardiac arrest within the prior 6 months	14
11. New York Heart Association class III or IV heart failure	68
12. Ejection fraction <40%	130
13. Pulmonary arterial hypertension treated with inhaled or intravenous pulmonary hypertension-specific therapy	1
14. Unsafe for participation as determined by a clinical nurse or investigative team	23
15. Does not own an Android or iPhone with study supported operating software	13
16. Not proficient in English (to enable informed consent, completion of study-related surveys, and compliance with study messages)	21
17. Wrist too large to wear a smartwatch comfortably.	0

Eligibility based on the first 14 criterion initially determined using the electronic medical record. If patients were deemed potentially eligible, they were contacted by phone and their eligibility based on the final 3 criteria was assessed. [a]N adds to greater than 518 as participants may meet more than one exclusion criterion.

Supplementary Table 5: Sample Activity and Exercise Planning Text Messages

Activity Message	Time	Day of Week	Environment	Duration	Other
Quick, look at the clock! ⏰ If the hour is odd, do that many toe-touches: stand ➡️ reach to the ceiling ➡️ reach to your toes ➡️ repeat.	All	Weekend or weekday	Indoor or Outdoor	Maintenance Completion	Emoji
What a morning! 🌇 [NAME], how about treating yourself to some beautiful sights by taking a short walk to start your day?	Morning	Weekend or weekday	Outdoor	Initiation Completion	Emoji Personalization
[NAME], you have worked so hard for the past months. Your progress here [HYPERLINK] is proof. What can you do today to be consistent?	Morning Lunch	Weekend or weekday	Indoor or Outdoor	Completion	Hyperlink Personalization

Exercise Planning Messages	Season	Duration in Cardiac Rehabilitation	Other
Remember how you felt when you started cardiac rehabilitation? Challenge yourself with exercise routines that will make you proud of yourself!	All	Maintenance Completion	Gain-framing
There are many ways to exercise while being mindful of your aches and pains. Water sports 🏊 may reduce the strain on your joints while also elevating your heart rate. ❤️	All	Maintenance Completion	Emoji
Set an exercise goal for tomorrow [NAME]. Short bursts of higher intensity exercise will help increase your heart rate. Try 3 minutes of lighter intensity exercise followed by 2 minutes of more vigorous exercise.	All	Initiation Completion	Personalization

Activity messages were tailored based on weather, day of week, time of day, and duration within the study, correlating with phase in cardiac rehabilitation (ie, initiation [0-30 days], maintenance [31-120 days], completion [121-182 days] phases). Exercise planning messages were tailored based on season and duration within the study.

Supplementary Table 6: Completed 6-Minute Walk Tests by Timepoint

	Total complete	Completed correctly within 7-days	Completed correctly within 30-days	Excluded 6-minute walk (<100 meters)	Reassigned 6-minute walk	Completed reassigned 6-minute walk within 30-days
**Baseline**	219	174	202	12	17	13
**3-months**	196	139	182	9	9	8
**6-months**	186	118	172	11	5	4

Participants were given up to 30-days to complete 6-minute walk tests after they were assigned at enrollment and at 3- and 6-months. Distances of less than 100 meters were excluded *a priori* from analysis given invalid data concerns.

Supplementary Figure 1: Effects of the Intervention on Change in 6-Minute Walk Distance at 6-months for Important Subgroups in Apple Watch (Top) and Fitbit (Bottom) Participants

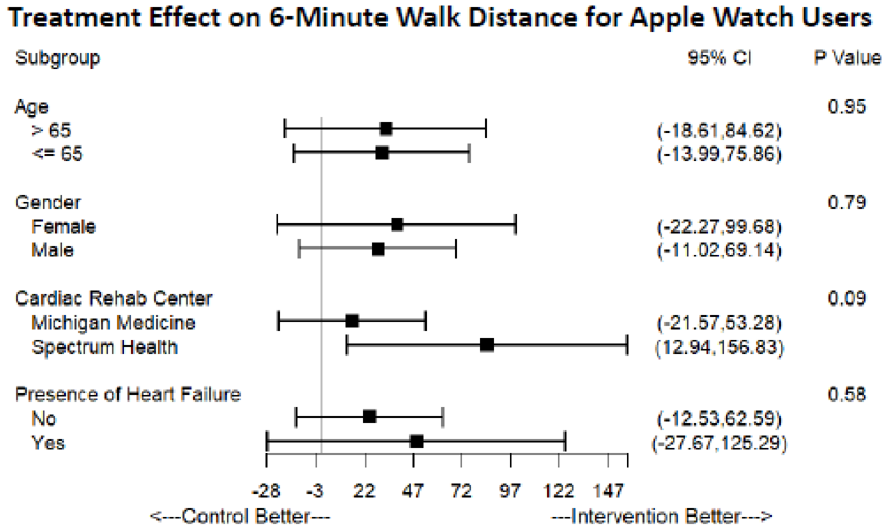

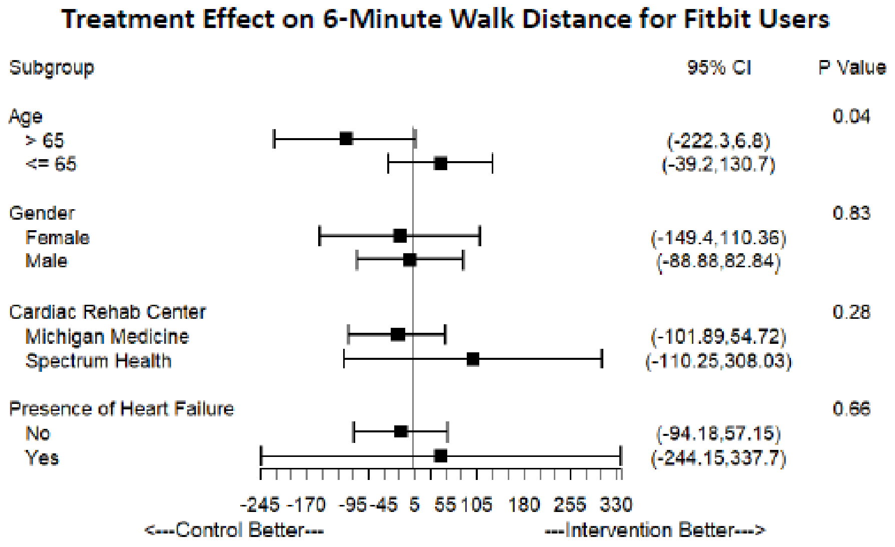

P-values for each subgroup correspond to the significance level of the interaction term between the intervention and the subgroup indicator based on linear regression analyses. Error bars refer to 95% confidence intervals.

Supplementary Figure 2: VALENTINE Mobile Application.

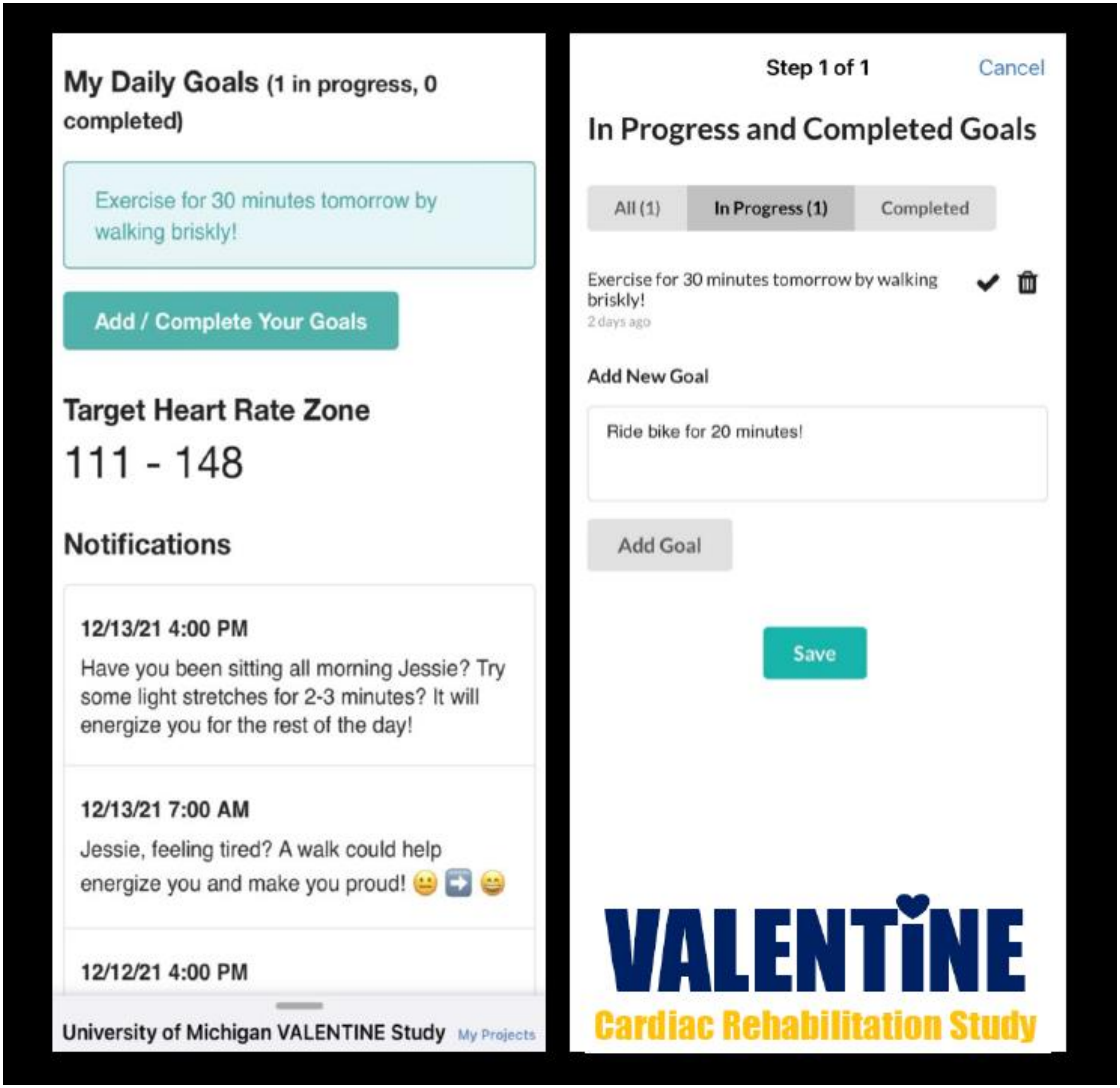

Supplement: Supplementary file 1 — Supplemental material [file 41746_2023_921_MOESM1_ESM.pdf]
